# Factors associated with *Anaplasma* spp. seroprevalence among dogs in the United States

**DOI:** 10.1186/s13071-016-1431-7

**Published:** 2016-03-22

**Authors:** Christopher S. McMahan, Dongmei Wang, Melissa J. Beall, Dwight D. Bowman, Susan E. Little, Patrick O. Pithua, Julia L. Sharp, Roger W. Stich, Michael J. Yabsley, Robert B. Lund

**Affiliations:** Department of Mathematical Sciences, Clemson University, Clemson, SC USA; IDEXX Laboratories, Inc., Westbrook, ME USA; College of Veterinary Medicine, Cornell University, Ithaca, NY USA; Department of Veterinary Pathobiology, Oklahoma State University, Stillwater, OK USA; Veterinary Medical Teaching Hospital, University of Missouri, Columbia, MO USA; Department of Veterinary Pathobiology, University of Missouri, Columbia, MO USA; Department of Population Health, University of Georgia, Athens, GA USA

**Keywords:** *Anaplasma platys*, Canine anaplasmosis, Seroprevalence, *Anaplasmaphagocytophilum*, Seroepidemiologic Studies, Ticks, United States, Zoonoses

## Abstract

**Background:**

Dogs in the United States are hosts to a diverse range of ticks and tick-borne pathogens, including *A. phagocytophilum*, an important emerging canine and human pathogen. Previously, a Companion Animal Parasite Council (CAPC)-sponsored workshop proposed factors purported to be associated with the infection risk for tick-transmitted pathogens in dogs in the United States, including climate conditions, socioeconomic characteristics, local topography, and vector distribution.

**Methods:**

Approximately four million test results from routine veterinary diagnostic tests from 2011–2013, which were collected on a county level across the contiguous United States, are statistically analyzed with the proposed factors *via* logistic regression and generalized estimating equations. Spatial prevalence maps of baseline *Anaplasma* spp. prevalence are constructed from Kriging and head-banging smoothing methods.

**Results:**

All of the examined factors, with the exception of surface water coverage, were significantly associated with *Anaplasma* spp. prevalence. Overall, *Anaplasma* spp. prevalence increases with increasing precipitation and forestation coverage and decreases with increasing temperature, population density, relative humidity, and elevation. Interestingly, socioeconomic status and deer/vehicle collisions were positively and negatively correlated with canine *Anaplasma* seroprevalence, respectively. A spatial map of the canine *Anaplasma* hazard is an auxiliary product of the analysis. *Anaplasma* spp. prevalence is highest in New England and the Upper Midwest.

**Conclusions:**

The results from the two posited statistical models (one that contains an endemic areas assumption and one that does not) are in general agreement, with the major difference being that the endemic areas model estimates a larger prevalence in Western Texas, New Mexico, and Colorado. As *A. phagocytophilum* is zoonotic, the results of this analysis could also help predict areas of high risk for human exposure to this pathogen.

**Electronic supplementary material:**

The online version of this article (doi:10.1186/s13071-016-1431-7) contains supplementary material, which is available to authorized users.

## Background

Dogs are susceptible to infection to numerous tick-borne rickettsial pathogens including *Anaplasma phagocytophilum,* the etiologic agent of granulocytic anaplasmosis in people, dogs, horses, sheep and other animals [1]. A closely related pathogen, *A. platys*, causes infectious cyclic thrombocytopenia in dogs and cross-reacts with antibodies to *A. phagocytophilum*. Clinical signs of canine granulocytic anaplasmosis range in severity, but commonly include fever, thrombocytopenia, lethargy, and polyarthritis, while infectious cyclic thrombocytopenia, caused by *A. platys,* is generally considered a mild disease except when co-infection exacerbates other diseases such as ehrlichiosis [2]. People with *A. phagocytophlium* infections may have flu-like symptoms, but rashes are rare, unlike other tick-borne zoonoses such as Lyme disease or Rocky Mountain spotted fever [3]. Although considered a low risk for human infection, a recent case report suggested *A. platys* might also be zoonotic [4].

In the United States, *Ixodes scapularis* (the blacklegged tick) and *Ixodes pacificus* (the western blacklegged tick) are considered the primary vectors of *A. phagocytophilum. Ixodes scapularis* is found in at least 32 states in the eastern and central states, while *I. pacificus* appears limited to five western states [5], but evidence of autochthonous transmission of pathogenic strains of *A. phagocytophilum* to people and dogs has only been documented in the Northeast, Upper Midwest, and limited parts of the western United States [6]. *Ixodes scapularis* and *Ixodes pacificus* are also found northward into Canada. In contrast, *Rhipicephalus sanguineus* (the brown dog tick) is thought to transmit *A. platys*, although this cycle has not been confirmed in North America. The distribution of *R. sanguineus* is described as cosmopolitan, as these ticks can infest buildings in otherwise inhospitable climes [7]. Brown dog ticks also thrive in arid areas with high temperatures. Accordingly, populations of this tick are most intense and infestations of premises are more common in the southern United States.

Transmission by tick vectors is considered the primary means of canine exposure to *Anaplasma* spp., thus variation in regional risk factors is tied to presence and abundance of competent tick vectors and vertebrate reservoirs. Factors associated with the presence of tick vectors include vector amplification hosts, pathogen reservoir host population densities, climate, and topography [8, 9]. Advances in testing and recording technologies have led to large datasets of diagnostic test results by county for canine exposure to *Anaplasma* spp. [6, 10]. With support from a veterinary diagnostic company (IDEXX Laboratories, Inc., Westbrook, ME), the Companion Animal Parasite Council (CAPC) has compiled a dataset of diagnostic test results that were reported by veterinary practitioners and a network of reference laboratories within the contiguous United States. This database allowed us to conduct the first comprehensive risk factor study of canine *Anaplasma* spp. in North America. The CAPC also convened a workshop to identify factors that are putatively associated with canine seroprevalence of tick-borne pathogens, specifically focusing on risk factors for which data are available, so these factors could be quantitatively evaluated for predictive power with respect to spatial-temporal seroprevalence patterns [11]. The objectives of this investigation were to identify risk factors associated with canine seroprevalence of *Anaplasma* spp. and to incorporate these factors into a refined spatial-temporal analysis. These data allow for the creation of maps that indicate risk of *Anaplasma* infections of people, dogs, horses, and other wildlife.

## Methods

### Data collection

To spatially analyze the canine seroprevalence of *Anaplasma* spp., the results of 3,950,852 diagnostic tests performed during 2011–2013 were acquired by the CAPC from IDEXX Laboratories, who provided qualitative (positive/negative) results reported for each county in the contiguous United States. Test results were generated using SNAP® 4Dx® and SNAP® 4Dx® Plus Test kits (IDEXX Laboratories, Inc.) which are point-of-care ELISAs to detect antigen from or antibodies to several vector-borne pathogens. The tests were performed at both the clinic level and at reference laboratories. The performance of these test kits was reported elsewhere [12, 13]. The *Anaplasma* portion of these tests uses a synthetic peptide from a major surface protein of *A. phagocytophilum* (MSP2/P44) and detects antibodies to both *A. phagocytoyphilum* and *A. platys* [13].

### Data analysis

#### Spatial structure of canine exposure to *Anaplasma* spp. in the United States

Two statistical smoothing techniques were applied to the data to generate a spatial prevalence map of canine exposure to *Anaplasma* spp. in the United States. A weighted head-banging algorithm was first used to reveal patterns in the data [14, 15]. To account for counties not reporting data, kriging, an interpolation method, was subsequently used to construct a spatially complete map [16].

#### Risk factors

Previously, 15 posited risk factors were proposed for canine exposure to pathogens transmitted by *I. scapularis, I. pacificus* or *R. sanguineus* [11]. Of these, nine were analyzed for predictive power in explaining the observed regional canine seroprevalence. To be considered, a factor had to be quantifiable with currently available data; this limited the number of factors to climate (annual temperature, precipitation, and relative humidity), socioeconomic characteristics (human population density and household income), and local topography (surface water, forestation coverage, and elevation) [11]. Finally, nationwide county-level deer densities were not available; hence, a state-by-state estimated annual probability of deer/vehicle collisions was used as a surrogate risk factor [17]. Counties within a state were assigned the collision proportion for the entire state (Additional file 1: Figure S1). The premise was that regions with greater deer/vehicle collision reports support higher deer populations. A list of the considered factors and their sources is provided in Table 1.

**Table 1:** Candidate factors, considered in both the Endemic Regions and Contiguous US models, along with their units, data sources, and spatial resolution

Category	Factor(s)	Scale	Source
Climate	Annual temperature (F)	Division	National Climate Data Center (NCDC)
	Annual precipitation (in)	Division	NCDC
	Annual relative humidity (%)	Station	NCDC
Geographic	Elevation (ft)	County	http://www.cohp.org/
	Percentage forest coverage (%)	County	United States Department of Agriculture (USDA)
	Percentage surface water coverage (%)	County	US Census Bureau
Societal	Population density (persons per square mile)	County	US Census Bureau
	Median household income ($)	County	US Census Bureau
Prostriate Tick Amplification	Deer/vehicle collisions (probability)	State	State Farm Insurance Company

#### Statistical methods

To assess the significance of the putative risk factors, let *Y*_*i*,*j*_ denote the number of positive tests in the *i*^*th*^ county during the *j*^*th*^ year and *n*_*i*,*j*_ the corresponding total number of tests performed. An estimate of the *i*^*th*^ county’s prevalence over the three study years is

$$ {\widehat{p}}_i=\left({Y}_{i,1}+{Y}_{i,2}+{Y}_{i,3}\right)/\left({n}_{i,1}+{n}_{i,2}+{n}_{i,3}\right). $$

Generalized linear models (GLMs) are used here with assumptions that the observed data are (1) independent and (2) follow a distribution belonging to an exponential family. For further details, see [18]. Here, it is assumed that the number of positive test results is a true random sample, obeying a binomial distribution (an exponential family member). Possible departures from this assumption are discussed later in the Conclusions. Consequently, a GLM can be formulated as

$$ \mathit{\mathsf{g}}\left({p}_{ij}\right)={\beta}_0+{{\displaystyle {\sum}_{k=1}^p{\beta}_kX}}_{ijk}={X}_{i^{\prime }j}\beta, $$

where *g* is an invertible link function, *X*_*ij*_ = (1, *X*_*ij*1_, …, *Xi*_*jp*_)′ is a vector of risk factors from the *i*^*th*^ county during the *j*^*th*^ year, and *β* = (*β*_0,…,_*β*_*p*_)′ is a vector of regression coefficients. Herein, *g* is specified to be the logistic link; i.e., $\mathit{\mathsf{g}}\left({p}_{ij}\right)= \log \left\{{p}_{ij}/\left(1-{p}_{ij}\right)\right\}.$ Models of this form are easily fit using standard statistical software. For a fixed county, it is unreasonable to assume that seroprevalence estimates are statistically independent in time. In fact, in endemic areas, infections persist in reservoir host populations; consequently, the number of positive test results from year-to-year in a given county may be highly positively correlated.

To allow for temporal correlation, a generalized estimating equation (GEE) was used to estimate regression coefficients [19, 20]. GEEs are similar in form to GLMs, but account for the correlation between observations within a particular county over time by minimizing a “weighted” sum of squares to obtain parameter estimators [19, 20] (GLMs minimize an “unweighted” sum of squares). To apply the GEE method a working correlation matrix has to be specified; e.g., independent, exchangeable, auto-regressive, etc. The specification of this matrix accounts for the temporal correlation within a given county. In order to prevent misspecification, an unstructured working correlation matrix was considered and its components were estimated along with the regression parameters. GEE models can be fitted using standard statistical software (e.g., SAS, Stata, Splus, and R) [21, 22].

While GEE techniques account for temporal dependence within a county, they assume observations from different counties are independent. Consequently, the weighted head-banging and Kriging algorithms [23, 24], which implicitly account for spatial dependence, were used to graphically display prevalence estimates. The weighted head-banging algorithm, which made use of 20 triples, was first used to smooth the county-level prevalence estimates. The weights were set as the reciprocal of the estimated standard deviation of the prevalence estimates. Thus, counties with more observations had more importance in the smoothing. Kriging was then applied to the head-banging estimates to infill counties not reporting data and to generate spatially complete prevalence maps. Kriging was implemented using the default settings within ArcGIS. Two main effects models, described below, were considered.

In describing model fits, estimated regression coefficients and their standard errors were obtained by fitting the proposed model in SAS. In order to retain model interpretability, this analysis considers only first-order models. Backward elimination was implemented, with a cutoff of 0.05, to complete model selection; i.e., the factor with the highest p-value greater than 0.05 was removed from the model at each step. Based on variance inflation factors, it was found that multicollinearity was not a significant issue. From these statistics, confidence intervals were constructed. To assess the quality of the model fit, a coefficient of determination, *R*^2^, is reported [25].

### Endemic region and contiguous US models

Two models were posited. The first was an “Endemic Regions” model and only used data from regions where *A. phagocytophilum* was considered potentially endemic based on published reports and expert opinion (shown in Additional file 2: Figure S2). Although data to indicate a particular region is endemic are imprecise, we subsequently show that the conclusions are not heavily dependent on this region’s definition. The second model considered was a “Contiguous US" model. Here, an indicator factor was added that demarcated whether or not a county was located within the *A. phagocytophilum-*endemic area (Additional file 2: Figure S2). This latter approach made use of all available data.

## Results and discussion

### Spatial prevalence

**Fig. 1:**
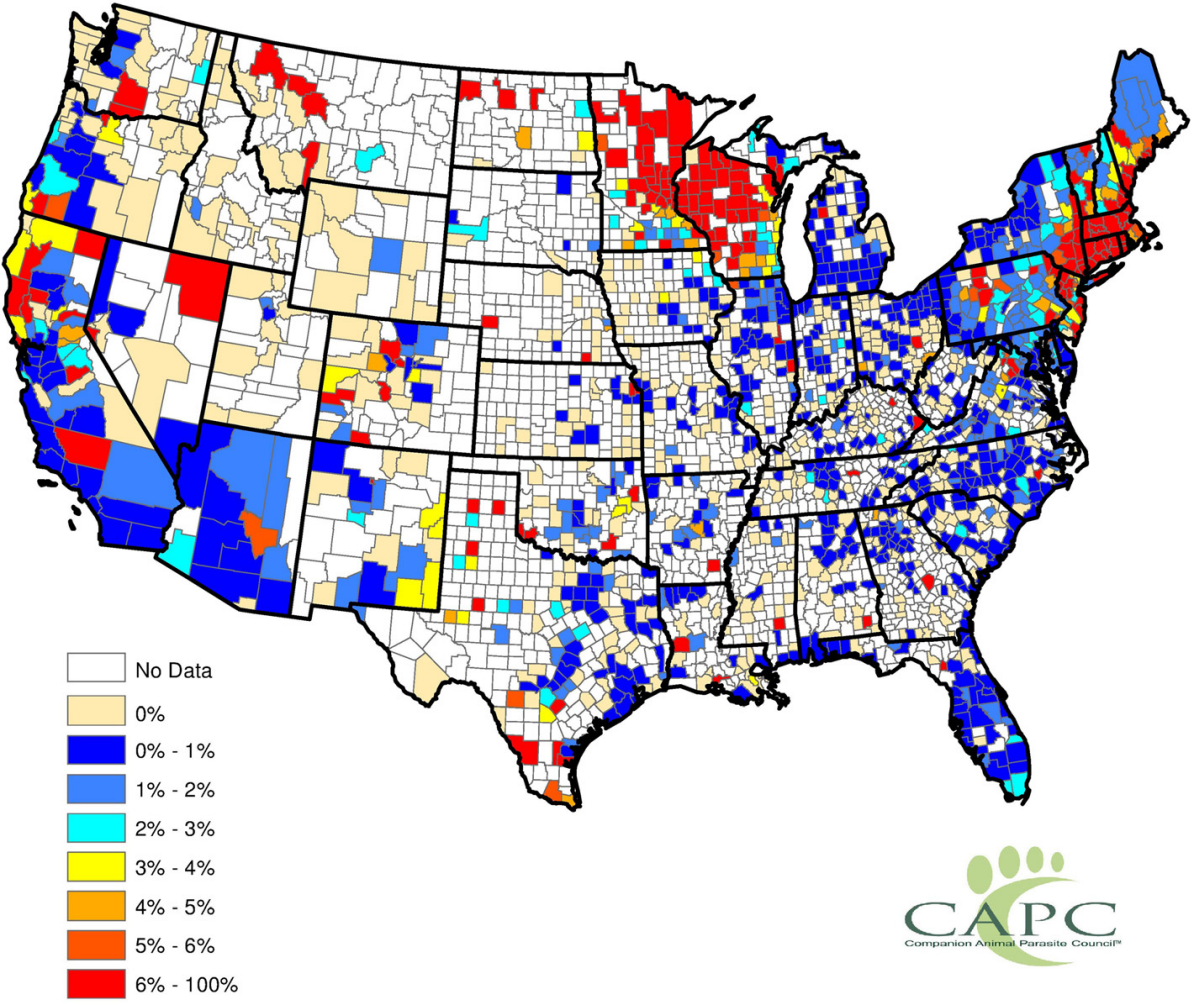
Map illustrating percentages of positive tests for canine exposure to *Anaplasma* spp. reported from US counties from 2011 to 2013

Nationwide, from 2011–2013, 3.76 % of tests were seropositive (4.26 % in 2011, 4.45 % in 2012, and 3.24 % in 2013). Approximately 1,500 of 3,144 US counties reported data each year, although this number varied slightly from year-to-year. Figure 1 shows the distribution and prevalence of dogs with antibodies to *Anaplasma* spp. by county. Most *Anaplasma*-positive test results originated from the Upper Midwest and Northeast, with the highest probabilities coming from northern Wisconsin, northern Minnesota, and eastern New England. Most counties not reporting data are in regions where these infections are considered uncommon (e.g., the South, Southwest and West), with the exception of the Rio Grande River Valley north through eastern New Mexico and Colorado.

**Fig. 2:**
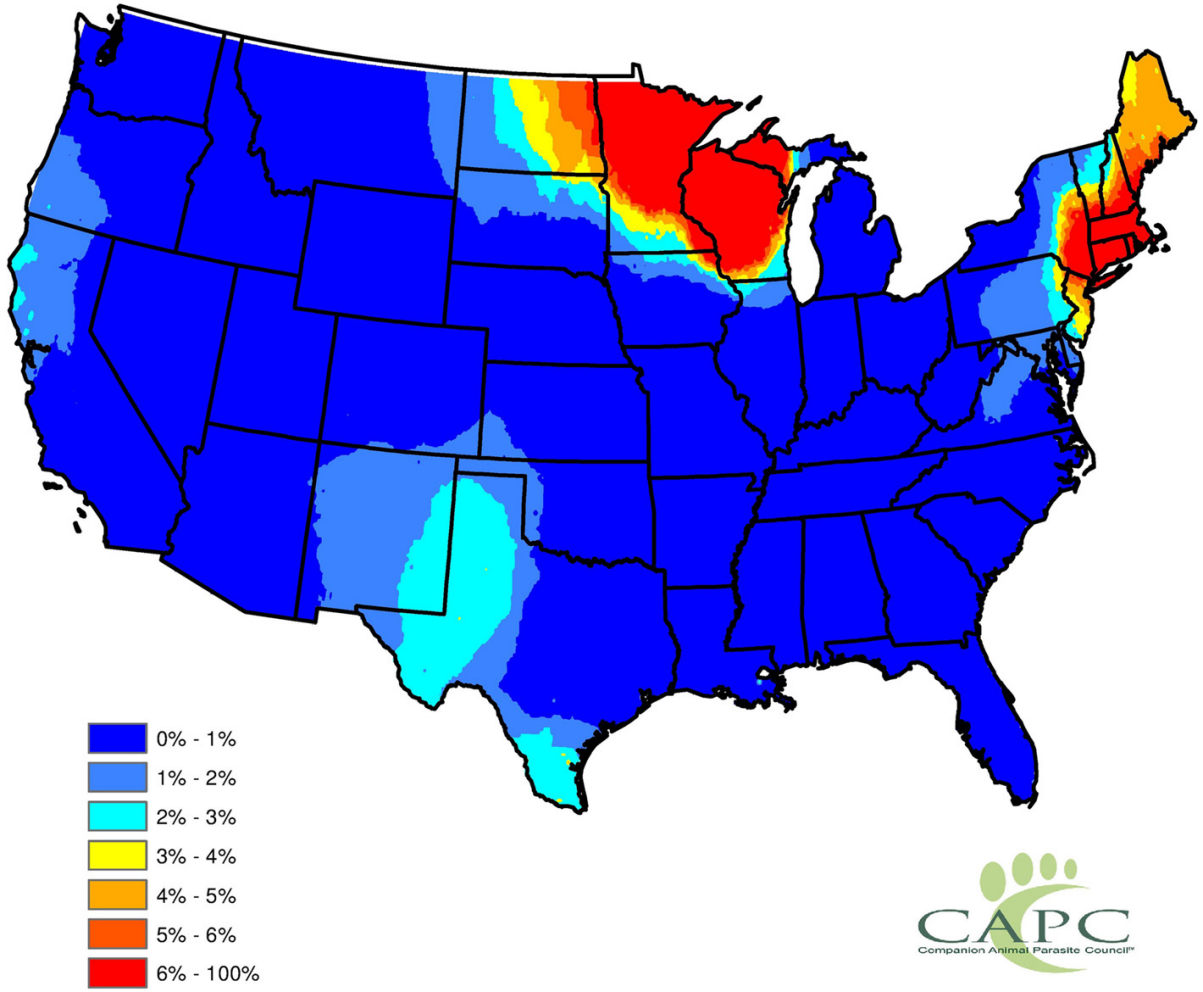
Statistically Smoothed Prevalence Estimates for Canine Exposure to *Anaplasma* spp. (2011 to 2013). Spatial smoothing was completed *via* the head-banging and Kriging algorithms

Prevalence was highly variable and data were missing for many counties, thus, to improve map utility, these estimates were statistically smoothed using head-banging and kriging algorithms. The expected prevalence of canine exposure to *Anaplasma* spp. during a typical year by county is shown in Fig. 2. These data confirm that canine exposure to *Anaplasma* spp. was most prevalent in the Northeast, upper Midwest, northern California, and western Texas and eastern New Mexico.

### Risk factor data

Several factors were significantly associated with the prevalence of *Anaplasma*-positive dogs, although the significant factors slightly change between the Endemic Regions and Contiguous US models (Table 2). All factors except for water coverage were significant with 95 % confidence in the Contiguous US model. When just the endemic regions were considered, all factors except water coverage and elevation were significant with 95 % confidence. Temperature, population density, relative humidity, elevation, and deer vehicle collisions are negatively correlated with *Anaplasma* prevalence and precipitation, forestation coverage, and median household income are positively correlated with *Anaplasma* prevalence.

**Table 2:** Estimates, standard errors, and odds ratios for the parameters corresponding to the factors found to be significantly associated with prevalence of canine exposure to *Anaplasma* spp. See Table 1 for the factor units

	Regression Coefficient Estimate	Standard Error	Exp(estimate)^a^ (Odds Ratio)	95 % CI^b^
Endemic Regions model				
Intercept	1.2930	0.5858	3.6437	(1.1542, 11.5041)
Temperature	−0.0740	0.0067	0.9287	(0.9165, 0.9410)
Median household income	0.0192	0.0021	1.0194	(1.0152, 1.1024)
Population density	−0.0110	0.0050	0.9891	(0.9792, 0.9979)
Precipitation	0.0463	0.0200	1.047	(1.010, 1.0925)
Relative humidity	−0.0291	0.0057	0.9713	(0.9605, 0.9821)
Forest coverage	0.0780	0.0124	1.0811	(1.055, 1.1078)
Deer/Vehicle collision	−0.8158	0.0889	0.4423	(0.3715, 0.5266)
Contiguous US model				
Intercept	−0.1728	0.5849	0.8413	(0.2671, 2.6501)
Temperature	−0.0659	0.0056	0.9362	(0.9260, 0.9466)
Median household income	0.0197	0.0018	1.0200	(1.0163, 1.0236)
Population density	−0.0130	0.0043	0.9871	(0.9998, 1.0000)
Precipitation	0.0432	0.0165	1.0441	(1.0108, 1.0785)
Relative humidity	−0.0282	0.0050	0.9722	(0.9629, 0.9816)
Forest coverage	0.0708	0.0104	1.0734	(1.0517, 1.0954)
Deer/Vehicle collision	−0.8483	0.0780	0.4281	(0.3660, 0.5008)
Elevation	−0.0522	0.0240	0.9491	(0.9055, 0.9950)
Endemic/Non-endemic	1.2196	0.1473	3.3858	(2.5363, 4.5195)

^a^The Exp (estimate) column shows the estimated odds ratios

^b^The CI column gives a 95% confidence interval for the odds ratios. Intervals not containing unity imply that the factor is significant at the 0.05 level

There was a significant correlation in the prevalence of *Anaplasma* spp. in dogs between years, regardless of the model (Table 3). The highly positive correlations imply that regions experiencing high or low canine seroprevalence will likely experience similarly high or low proportions in the near future. Correlations between proportions two years apart were lower than those separated by one year.

**Table 3:** Estimated year-to-year working correlation matrix in each model

	2011	2012	2013
Endemic Regions model			
2011	1.0000	0.8966	0.8256
2012	0.8966	1.0000	0.8345
2013	0.8256	0.8345	1.0000
Contiguous US model			
2011	1.0000	0.8649	0.7407
2012	0.8649	1.0000	0.8032
2013	0.7407	0.8032	1.0000

#### Regional prevalence based on contiguous US and endemic regions models

**Fig. 3:**
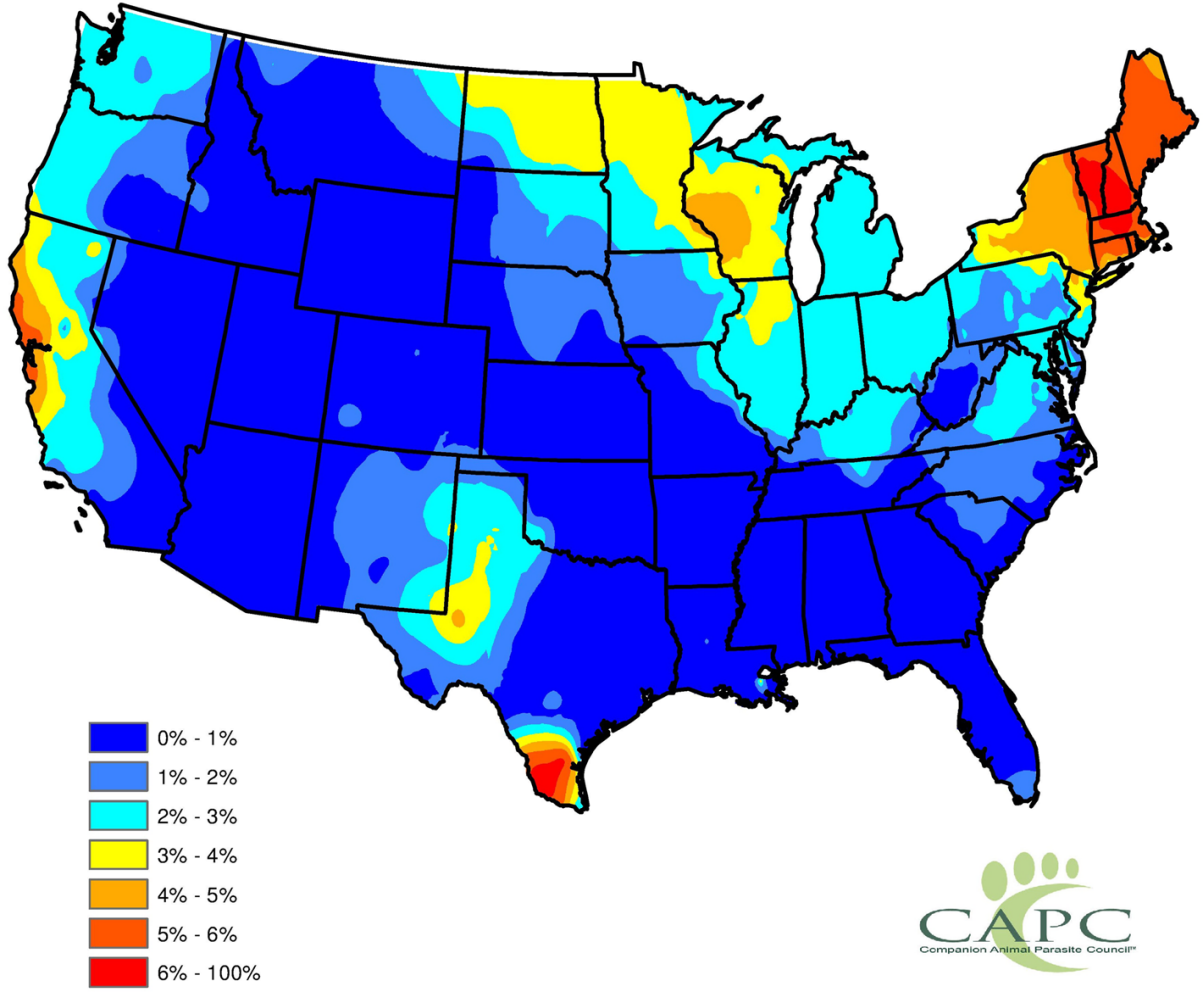
Estimated Canine *Anaplasma* Prevalence from Endemic Region Model. The presented results consist of statistically smoothed prevalence estimates, where the prevalence estimates were obtained from the fitted Endemic Region model. Spatial smoothing was completed *via* the head-banging and Kriging algorithms

**Fig. 4:**
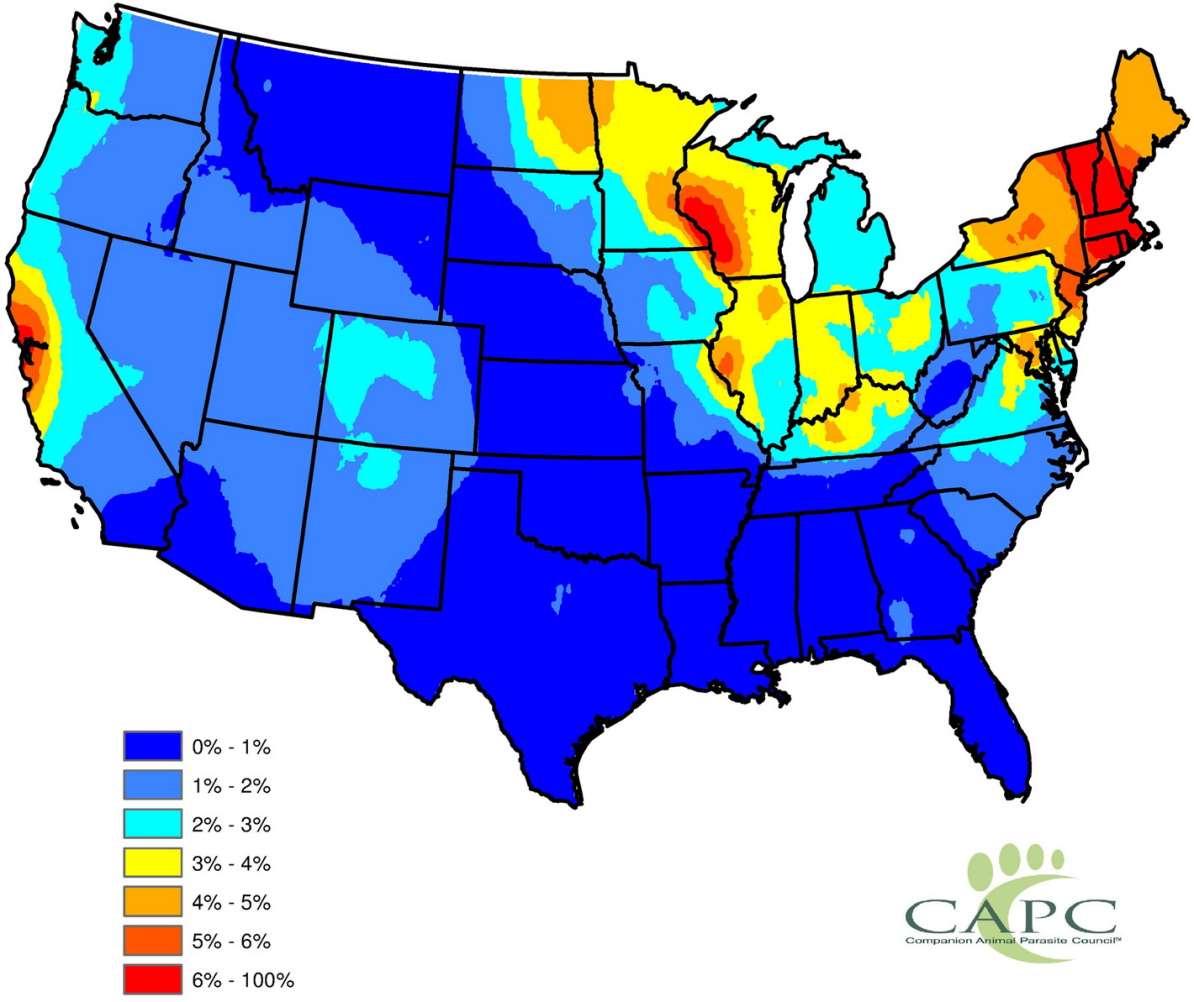
Estimated Canine *Anaplasma* Prevalence from Contiguous US Model. The presented results consist of statistically smoothed prevalence estimates, where the prevalence estimates were obtained from the fitted Contiguous US model. Spatial smoothing was completed *via* the head-banging and Kriging algorithms

Based on the Endemic Regions model, the highest prevalence estimates were reported for the Northeast followed by the upper Midwest, western Texas and central coastal California (Fig. 3). The Contiguous US model estimated higher prevalence in the upper Midwest but lower prevalence in Texas (Fig. 4). The model fits are summarized in Table 2. For the Endemic Regions model, prevalence estimates for counties in the endemic region were obtained from the fitted GEE model. This fit only uses data and factors for counties in the endemic regions. However, non-endemic regions were assigned the crude estimates depicted in Fig. 1 to coincide with the usual notion of prevalence (there are sporadic cases in non-endemic regions and some dogs also travel). The fitted models were similar and explain considerable structure: *R*^*2*^ for the fits are 0.72 (Endemic Regions model) and 0.71 (Contiguous US model).

## Conclusions

Like other tick-borne diseases in the United States, the incidence of human anaplasmosis has been increasing [26, 27]. Although canine anaplasmosis is not reportable, the incidence of seropositive canine cases also appears to be increasing. Similar to Bowman et al. [6], we found the highest prevalence of *Anaplasma* antibodies in dogs from the upper Midwest and eastern New England. These data also correlated with areas where the highest incidence of human anaplasmosis were reported, supporting the suggestion that dogs can make useful sentinels for human risk [26, 27]. Many of the dogs with antibodies reactive to *Anaplasma* are likely due to infection with *A. phagocytophilum*, given the general distribution and concordance with antibodies to *Borrelia burgdorferi* in dogs and human Lyme disease cases [6, 26, 28]. Further support comes from Qurollo et al. [29],who used *A. platys*- and *A. phagocytophilum*-specific assays to find similarly low seroprevalence of both pathogens in the Southeast and West. In contrast, the prevalence of antibodies to *A. phagocytophilum* was significantly higher in other regions. But, notably, there were isolated areas that had unexpectedly high prevalence estimates for *Anaplasma* (e.g., Texas, New Mexico, and Oklahoma) where neither *A. phagocytophilum* nor known tick vectors are common. Possible explanations of these findings include (1) exposure to *A. platys* or a novel *Anaplasma* spp., (2) an unrecognized novel *A. phagocytophilum* vector-reservoir transmission cycle in that region or (3) a relatively high frequency of dogs tested that had previously traveled to endemic regions [6]. These data, while sometimes enigmatic, should not be ignored as demonstrated by similar unexplained foci in the upper Midwest, where a novel *E. muris*-like agent was ultimately found in association with an unexpectedly high seroprevalence of *Ehrlichia* spp. among dogs [6, 30, 31].

Data from both the Endemic Regions and Contiguous US models agreed well with each other and original serologic data. However, there were some minor differences between the two models that resulted in some regions having a higher or lower estimated prevalence. For example, the Contiguous US model had higher prevalence estimates than the Endemic Regions model in some regions of the upper Midwest (e.g., Wisconsin, Minnesota, and Illinois) where granulocytic anaplasmosis is considered endemic and other regions of the Midwest (e.g., Indiana, Kentucky, and Ohio) where granulocytic anaplasmosis is currently considered rare. Also, the Contiguous US model estimated a lower prevalence for Maine, where granulocytic anaplasmosis is common. Lastly, the Contiguous US model estimated lower prevalence in western Texas, which was arguably influenced by smaller sample sizes.

The estimated regression coefficient for the endemic risk factor in the Contiguous US model is positive and significant. This implies higher prevalence among dogs living in areas where human granulocytic anaplasmosis is endemic.

Numerous factors were useful predictors for the seroprevalence of *Anaplasma* in dogs. Because rodents and white-tailed deer are important in the maintenance of *A. phagocytophilum* in nature, the association with increased forest coverage and decreased human population density is likely tied to suitable habitat for these critical wildlife species. Forest cover was also associated with higher prevalence of another tick-borne pathogen, *E. chaffeensis,* in white-tailed deer [32]. Importantly, forest fragmentation is highly associated with increasing Lyme disease incidence so these fragmented habitats will likely be important areas for *A. phagocytophilum*; however, the scale of this study was not fine enough to investigate edge effects [33].

Climatic variables such as temperature, precipitation and relative humidity have been associated with prevalence of ticks and tick-borne pathogens [34–36]. In both of our models, precipitation was positively associated with *Anaplasma* infections in dogs and temperature was negatively associated with prevalence. Although one previous study found no effect of precipitation on the density of *I. scapularis*, a more recent long-term study found that increased regional winter precipitation was associated with higher tick densities [37]. Ixodid tick survival and activity are tied to temperature, and a recent study found that *I. scapularis* survived better under temperatures more representative of northern states compared with those in the southern states [38]. Relative humidity is important for ixodid ticks to maintain moisture while off of the host, but both of our models found that increasing relative humidity was negatively associated with *Anaplasma* seroprevalence in dogs. A plausible explanation for this finding is that increased humidity may well be related to decreased tick densities. That is, higher humidity levels are conducive to mold and fungal growth to which ticks are fatally susceptible to as eggs and during molting. For example, [39, 40] reported that *I.ricinus* densities on rodents decreased with increasing relative humidity.

The seroprevalence of *Anaplasma* spp. in dogs decreased as deer/vehicle collision reports increased, which was contrary to our initial hypothesis given the importance of deer to the life cycle of *I. scapularis* [41]. Unfortunately, this factor does not account for the rural/urban nature of the habitats or road types (e.g., secondary or tertiary) where the collisions take place; see [42] for a more in depth discussion of these issues. While further investigation is warranted to understand this negative association, other authors have also found “deer density associations” counter intuitive, see [32, 40, 43–46] for some of the discussion and related literature.

Another puzzling finding was the positive association of *Anaplasma* seroprevalence in dogs with increasing household income. It is conceivable that high *Anaplasma* spp. prevalence areas coincide with some of the richer areas of the United States, thus confounding the factor. While people in these richer areas may engage in behaviors that increase the likelihood of ticks feeding on their dogs, such as outdoor recreational activities, wealthier dog owners may tend to keep their pet predominantly indoors, thus minimizing their risk of acquiring ticks [47]. However, even dogs that spend only small periods of time outdoors can acquire vector-borne infections; thus, the use of tick preventives is recommended for all dogs. Dogs in poorer regions may never be taken to the vet, clearing the infection themselves or may be treated with antibiotics (and not tested). Overall, the confounding nature of socioeconomic status merits further study.

The fitted models explain much of the data, but better fits could be achieved by including additional factors. One difficulty is that these data may not have been a true random sample, with correlation existing between some of the tests conducted at the same location. A more problematic issue lies with sampling biases: dogs in different parts of the country may be tested for exposure to *Anaplasma* for different reasons. For example, veterinarians in the Upper Midwest and Northeast, where Lyme disease has a high prevalence, may be more likely to screen all dogs using this rapid test. However, in areas where canine anaplasmosis or Lyme disease is uncommon, it is possible that only dogs with clinical signs or with travel histories to endemic regions may be tested. Other dogs could be coincidentally tested when screened for other vector-borne pathogens (e.g., heartworm), as the SNAP 4Dx Plus Test simultaneously tests for four distinct pathogen genera. Diagnostic tests specific for exposure to *A. platys* and acquisition of travel histories of seropositive dogs could help answer these questions about areas where granulocytic anaplasmosis is not considered endemic. Unfortunately, such data were unavailable at the time of this study. Because of these issues, caution should be used when comparing prevalence at two different areas of the United States.

The spatial prevalence maps here should not be interpreted at too fine of a spatial scale, they are intended as rough guidance. A county’s estimated prevalence is impacted by factor conditions in that county and by factor conditions in adjacent counties. For example, ticks are not expected to be numerous within New York City (say Manhattan), even though our mathematical model does not predict zero prevalence for Manhattan. Due to the zoonotic nature of anaplasmosis, one may compare the findings of our analysis to the reported geographic distribution of anaplasmosis incidence in humans provided by the Centers for Disease Control and Prevention [48]. Further, as *I. scapularis* is a primary vector of anaplasmosis another relevant comparison can be made between our findings and the predicted geographic density of nymphal *I. scapularis* presented in [49]. From these comparisons, one will note that the geographic patterns of our spatial prevalence maps are largely in agreement with the spatial patterns found in these two surrogate measures.

Clearly, our list of risk factors is incomplete. Tick abundances, for example, are likely an important consideration, but these data are not available for the entire United States. However, this model can be updated as more factors such as tick densities, land-use changes, or acaricide use are obtained.

## Additional files

Additional file 1: Figure S1. Statewide Deer/Vehicle Collision Percentages in 2013. (JPG 2151 kb) Additional file 2: Figure S2. Granulocytic anaplasmosis-endemic areas of the United States. Areas where granulocytic anaplasmosis is considered endemic are reflective of counties surrounding established *I. scapularis* and *I. pacificus* populations and where clinical diagnosis of granulocytic anaplasmosis or competent reservoir hosts have been reported. Counties where granulocytic anaplasmosis is considered endemic are shaded red; non-endemic counties are shaded white. (JPG 2025 kb)

## References

[1] Little SE (2010). Ehrlichiosis and anaplasmosis in dogs and cats. Vet Clin North Am Small Anim Pract.

[2] Gaunt S, Beall M, Stillman B, Lorentzen L, Diniz P, Chandrashekar R, et al. Experimental infection and co-infection of dogs with *Anaplasma platys* and *Ehrlichia canis*: hematologic, serologic and molecular findings. Parasit Vectors. 2010;3:33. 10.1186/1756-3305-3-33 PMC2859368 20377870

[3] Bakken JS, Dumler JS, Chen SM, Eckman MR, Van Etta LL, Walker DH (1994). Human granulocytic ehrlichiosis in the upper Midwest United States. A new species emerging?. JAMA.

[4] Arraga-Alvarado CM, Qurollo BA, Parra OC, Berrueta MA, Hegarty BC, Breitschwerdt EB. Case report: Molecular evidence of *Anaplasma platys* infection in two women from Venezuela. Am J Trop Med Hyg. 2014;91:1161–5. 10.4269/ajtmh.14-0372 PMC4257640 25266347

[5] Dennis DT, Nekomoto TS, Victor JC, Paul WS, Piesman J. Reported distribution of *Ixodes scapularis* and *Ixodes pacificus* (Acari: Ixodidae) in the United States. J Med Entomol. 1998;35:629–38. 10.1093/jmedent/35.5.629 9775584

[6] Bowman D, Little SE, Lorentzen L, Shields J, Sullivan MP, Carlin EP. Prevalence and geographic distribution of *Dirofilaria immitis, Borrelia burgdorferi, Ehrlichia canis,* and *Anaplasma phagocytophilum* in dogs in the United States: results of a national clinic-based serologic survey. Vet Parasitol. 2009;160:138–48. 10.1016/j.vetpar.2008.10.093 19150176

[7] Dantas-Torres F. The brown dog tick, *Rhipicephalus sanguineus* (Latreille, 1806) (Acari: Ixodidae): from taxonomy to control. Vet Parasitol. 2008;152:173–85. 10.1016/j.vetpar.2007.12.030 18280045

[8] Simon JA, Marrotte RR, Desrosiers N, Fiset J, Gaitan J, Gonzalez A, et al. Climate change and habitat fragmentation drive the occurrence of *Borrelia burgdorferi*, the agent of Lyme disease, at the northeastern limit of its distribution. Evol Appl. 2014;7:750–64. 10.1111/eva.12165 PMC4227856 25469157

[9] Wimberly MC, Baer AD, Yabsley MJ (2008). Enhanced spatial models for predicting the geographic distributions of tick-borne pathogens. Int J Health Geogr.

[10] CAPC. Companion Animal Parasite Council Parasite Prevalence Maps. 2014 [cited; Available from: http://www.capcvet.org/parasite-prevalence-maps.

[11] Stich RW, Blagburn BL, Bowman DD, Carpenter C, Cortinas MR, Ewing SA (2014). Quantitative factors proposed to influence the prevalence of canine tick-borne disease agents in the United States. Parasit Vectors.

[12] Chandrashekar R, Mainville CA, Beall MJ, O'Connor T, Eberts MD, Alleman AR (2010). Performance of a commercially available in-clinic ELISA for the detection of antibodies against *Anaplasma phagocytophilum, Ehrlichia canis*, and *Borrelia burgdorferi* and *Dirofilaria immitis* antigen in dogs. Am J Vet Res.

[13] Stillman BA, Monn M, Liu J, Thatcher B, Foster P, Andrews B (2014). Performance of a commercially available in-clinic ELISA for detection of antibodies against *Anaplasma phagocytophilum, Anaplasma platys, Borrelia burgdorferi, Ehrlichia canis,* and *Ehrlichia ewingii* and *Dirofilaria immitis* antigen in dogs. J Am Vet Med Assoc.

[14] Wang D, Bowman DD, Brown HE, Harrington LC, Kaufman PE, McKay T (2014). Factors influencing U.S. canine heartworm (*Dirofilaria immitis*) prevalence. Parasit Vectors.

[15] Mungiole M, Pickle LW, Simonson KH (1999). Application of a weighted head-banging algorithm to mortality data maps. Stat Med.

[16] Deutsch CV, Journel AG. Geostatistical Software Library and User’s Guide (Applied Geostatistics Series). Oxford, England: Oxford University Press; 1992. p. 63–95.

[17] StateFarmInsCo. It's West Virginia Again. Mountain State Leads the State Farm® List Of States Where Deer-Vehicle Confrontations Are Most Likely. 2012. Available from: http://www.statefarm.com/aboutus/newsroom/2012/10/23/deer-vehicle-confrontations.

[18] McCullagh P, Nelder JA (1989). Generalized Linear Models.

[19] Ballinger GA (2004). Using Generalized Estimating Equations for Longitudinal Data Analysis. Organizational Research Methods.

[20] Liang K-Y, Zeger SL (1986). Longitudinal data analysis using generalized linear models. Biometrika.

[21] Horton NJ, Lipsitz SR (1999). Review of Software to Fit Generalized Estimating Equation Regression Models. The American Statistician.

[22] Ying G, Liu C (2006). Statistical Analysis of Clustered Data using SAS System.

[23] Stein ML (2012). Interpolation of spatial data: some theory for kriging.

[24] Hansen KM (1991). Head-banging: robust smoothing in the plane. IEEE Transactions Geoscience and Remote Sensing.

[25] Natarajan S, Lipsitz S, Parzen M, Lipshultz (2007). A measure of partial assciation for generalized estimating equations. Statistical Modelling.

[26] Dahlgren FS, Heitman KN, Drexler NA, Massung RF, Behravesh CB. Human Granulocytic Anaplasmosis in the United States from 2008 to 2012: A Summary of National Surveillance Data. Am J Trop Med Hyg. 2015;93:66–72. 10.4269/ajtmh.15-0122 PMC4497906 25870428

[27] Dahlgren FS, Mandel EJ, Krebs JW, Massung RF, McQuiston JH (2011). Increasing incidence of *Ehrlichia chaffeensis* and *Anaplasma phagocytophilum* in the United States, 2000–2007. Am J Trop Med Hyg.

[28] Herrmann JA, Dahm NM, Ruiz MO, Brown WM (2014). Temporal and Spatial Distribution of Tick-Borne Disease Cases among Humans and Canines in Illinois (2000–2009). Environ Health Insights.

[29] Qurollo BA, Chandrashekar R, Hegarty BC, Beall MJ, Stillman BA, Liu J, et al. A serological survey of tick-borne pathogens in dogs in North America and the Caribbean as assessed by *Anaplasma phagocytophilum, A. platys, Ehrlichia canis, E. chaffeensis, E. ewingii,* and *Borrelia burgdorferi* species-specific peptides. Infect Ecol Epidemiol. 2014;4:24699. http://dx.doi.org/10.3402/iee.v4.24699. 10.3402/iee.v4.24699 PMC4212082 25405006

[30] Pritt BS, Sloan LM, Johnson DK, Munderloh UG, Paskewitz SM, McElroy KM (2011). Emergence of a new pathogenic *Ehrlichia* species, Wisconsin and Minnesota, 2009. N Engl J Med.

[31] Telford Iii SR, Goethert HK, Cunningham JA (2011). Prevalence of *Ehrlichia muris* in Wisconsin Deer Ticks Collected During the Mid 1990s. Open Microbiol J.

[32] Yabsley MJ, Wimberly MC, Stallknecht DE, Little SE, Davidson WR (2005). Spatial analysis of the distribution of *Ehrlichia chaffeensis*, causative agent of human monocytotropic ehrlichiosis, across a multi-state region. Am J Trop Med Hyg.

[33] Tran PM, Waller L (2013). Effects of landscape fragmentation and climate on Lyme disease incidence in the northeastern United States. Ecohealth.

[34] Parham PE, Waldock J, Christophides GK, Hemming D, Agusto F, Evans KJ, et al. Climate, environmental and socio-economic change: weighing up the balance in vector-borne disease transmission. Philos Trans R Soc Lond B Biol Sci. 2015;370. http://dx.doi.org/10.1098/rstb.2013.0551. 10.1098/rstb.2013.0551 PMC4342957 25688012

[35] Randolph SE (1993). Climate, satellite imagery and the seasonal abundance of the tick *Rhipicephalus appendiculatus* in southern Africa: a new perspective. Med Vet Entomol.

[36] Randolph SE (2013). Is expert opinion enough? A critical assessment of the evidence for potential impacts of climate change on tick-borne diseases. Anim Health Res Rev.

[37] Hayes LE, Scott JA, Stafford 3rd KC. Influences of weather on *Ixodes scapularis* nymphal densities at long-term study sites in Connecticut. Ticks Tick Borne Dis. 2015;6:258–66. 10.1016/j.ttbdis.2015.01.006 25687504

[38] Ginsberg HS, Rulison EL, Azevedo A, Pang GC, Kuczaj IM, Tsao JI, et al. Comparison of survival patterns of northern and southern genotypes of the North American tick *Ixodes scapularis* (Acari: Ixodidae) under northern and southern conditions. Parasit Vectors. 2014;7:394. 10.1186/1756-3305-7-394 PMC4153913 25160464

[39] Boyard C, Vourc'h G, Barnouin J. The relationships between *Ixodes ricinus* and small mammal species at the woodland-pasture interface. Exp Appl Acarol. 2008;44:61–76. 10.1007/s10493-008-9132-3 18247140

[40] Kiffner C, Lodige C, Alings M, Vor T, Ruhe F. Attachment site selection of ticks on roe deer, *Capreolus capreolus*. Exp Appl Acarol. 2011;53:79–94. 10.1007/s10493-010-9378-4 PMC2992130 20585837

[41] Kilpatrick HJ, LaBonte AM, Stafford KC (2014). The relationship between deer density, tick abundance, and human cases of Lyme disease in a residential community. J Med Entomol.

[42] Hothorn T, Brandi R, Muller J (2012). Large-Scale Model-Based Assessmsnt of Deer-Vehicle Collision Risk. PLoS One.

[43] Rand PW, Lubelczyk C, Holman MS, Lacombe EH, Smith Jr RP. Abundance of *Ixodes scapularis* (Acari: Ixodidae) after the complete removal of deer from an isolated offshore island, endemic for Lyme Disease. J Med Entomol. 2004;41:779–84. 10.1603/0022-2585-41.4.779 15311475

[44] Jordan RA, Schulze TL, Jahn MB. Effects of reduced deer density on the abundance of *Ixodes scapularis* (Acari: Ixodidae) and Lyme disease incidence in a northern New Jersey endemic area. J Med Entomol. 2007;44:752–7. 10.1603/0022-2585(2007)44[752:eorddo]2.0.co;2 17915504

[45] Van Buskirk J, Ostfeld RS (1995). Controlling Lyme disease by modifying the density and species composition of tick hosts. Ecol Appl.

[46] Ostfeld RS, Canham CD, Oggenfuss K, Winchcombe RJ, Keesing F (2006). Climate, deer, rodents, and acorns as determinants of variation in lyme-disease risk. PLoS Biol.

[47] Des Vignes F, Piesman J, Heffernan R, Schulze TL, Stafford III KC, Fish D. Effect of tick removal on transmission of *Borrelia burgdorferi* and *Ehrlichia phagocytophila* by *Ixodes scapularis* nymphs. J Infect Dis. 2001;183:773–8. 10.1086/318818 11181154

[48] Centers for Disease Control and Prevention. Statistics and Epidemiology: Annual Cases of Anaplasmosisin the United States, Geography. 2015 [cited; Available from: http://www.cdc.gov/anaplasmosis/stats/#geography

[49] Diuk-Wasser MA, Vourc'h G, Cislo P, Hoen AG, Melton F, Hamer SA (2010). Field and climate-based model for predicting the density of host-seeking nymphal *Ixodes scapularis*, an important vector of tick-borne disease agents in the eastern United States. Glob Ecol Biogeogr.

