# Supplementary figures and images for: Factors associated with Anaplasma spp. seroprevalence among dogs in the United States
Source: Parasit Vectors. 2016 Mar 22;9:169. doi: 10.1186/s13071-016-1431-7 (PMC4804572; doi:10.1186/s13071-016-1431-7)

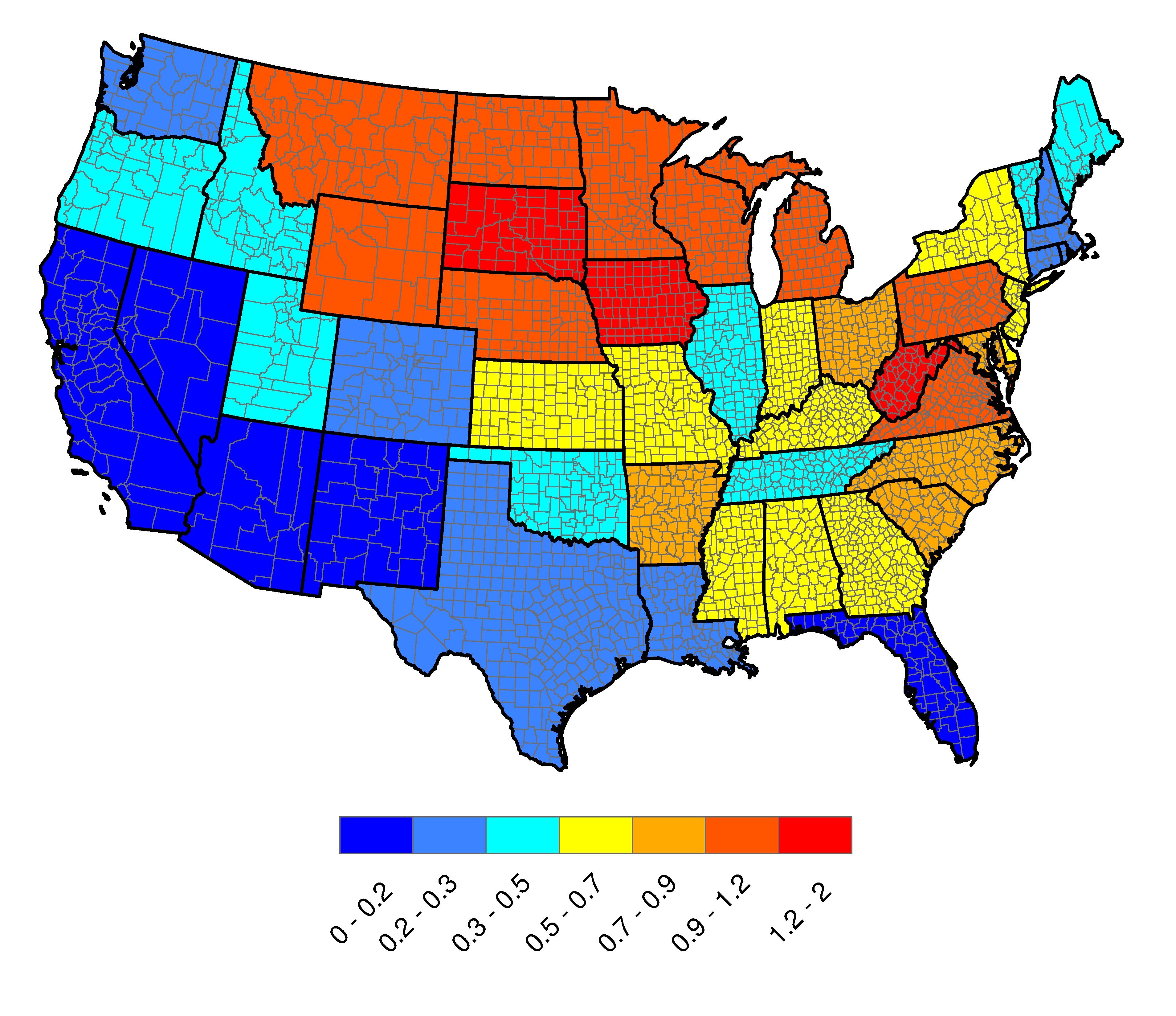

Supplement: Additional file 1: Figure S1. — Statewide Deer/Vehicle Collision Percentages in 2013. (JPG 2151 kb) [file 13071_2016_1431_MOESM1_ESM.jpg]

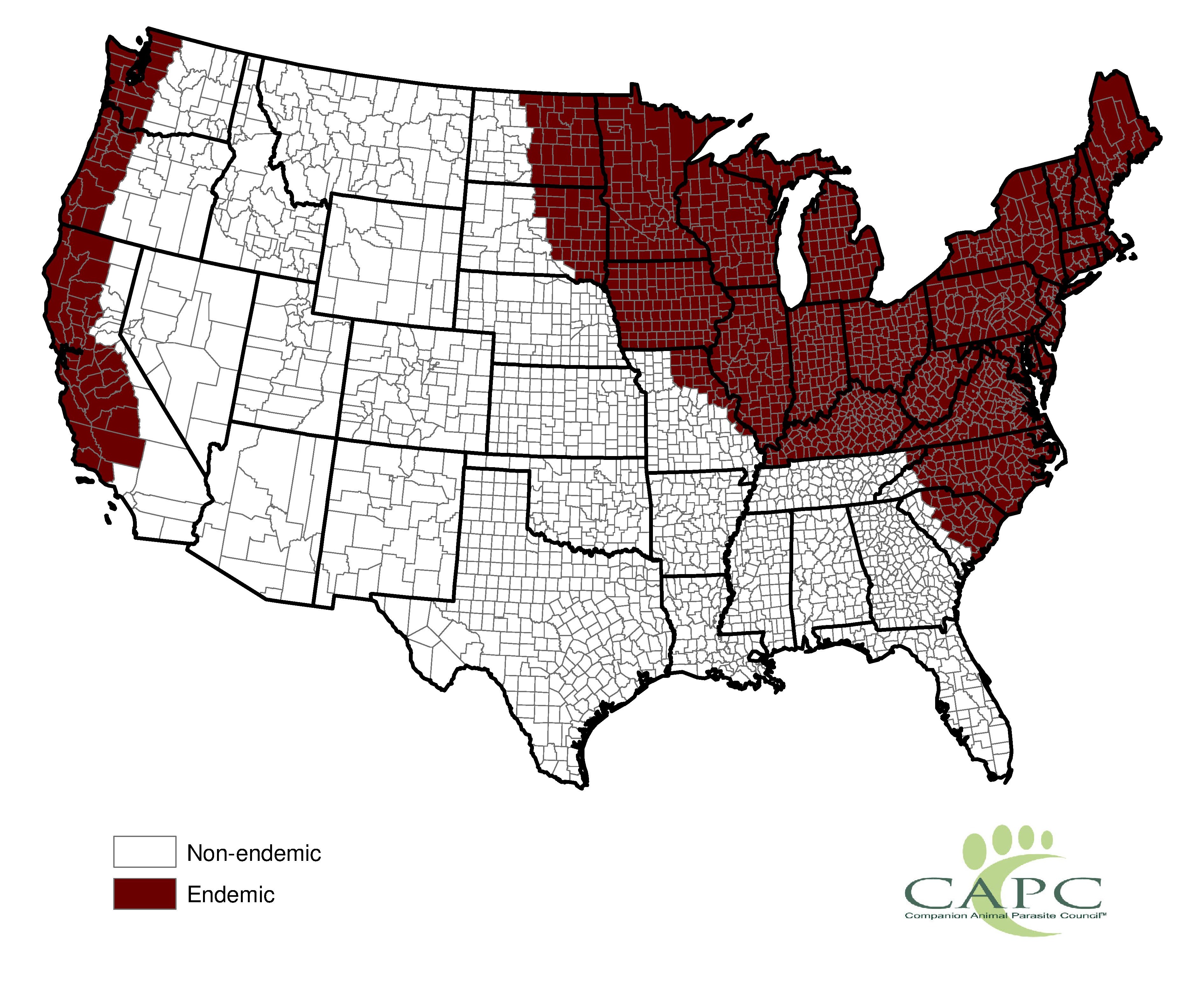

Supplement: Additional file 2: Figure S2. — Granulocytic anaplasmosis-endemic areas of the United States. Areas where granulocytic anaplasmosis is considered endemic are reflective of counties surrounding established I. scapularis and I. pacificus populations and where clinical diagnosis of granulocytic anaplasmosis or competent reservoir hosts have been reported. Counties where granulocytic anaplasmosis is considered endemic are shaded red; non-endemic counties are shaded white. (JPG 2025 kb) [file 13071_2016_1431_MOESM2_ESM.jpg]
